# Transcriptome analysis and differential gene expression profiling of two contrasting quinoa genotypes in response to salt stress

**DOI:** 10.1186/s12870-020-02753-1

**Published:** 2020-12-30

**Authors:** Pibiao Shi, Minfeng Gu

**Affiliations:** Xinyang Agricultural Experiment Station of Yancheng City, Yancheng, 224049 Jiangsu China

**Keywords:** RNA-Seq, Transcriptome analysis, Quinoa, Salt stress, Candidate genes, Differential expression

## Abstract

**Background:**

Soil salinity is one of the major abiotic stress factors that affect crop growth and yield, which seriously restricts the sustainable development of agriculture. Quinoa is considered as one of the most promising crops in the future for its high nutrition value and strong adaptability to extreme weather and soil conditions. However, the molecular mechanisms underlying the adaptive response to salinity stress of quinoa remain poorly understood. To identify candidate genes related to salt tolerance, we performed reference-guided assembly and compared the gene expression in roots treated with 300 mM NaCl for 0, 0.5, 2, and 24 h of two contrasting quinoa genotypes differing in salt tolerance.

**Results:**

The salt-tolerant (ST) genotype displayed higher seed germination rate and plant survival rate, and stronger seedling growth potential as well than the salt-sensitive (SS) genotype under salt stress. An average of 38,510,203 high-quality clean reads were generated. Significant Gene Ontology (GO) terms and Kyoto Encyclopedia of Genes and Genomes (KEGG) pathways were identified to deeper understand the differential response. Transcriptome analysis indicated that salt-responsive genes in quinoa were mainly related to biosynthesis of secondary metabolites, alpha-Linolenic acid metabolism, plant hormone signal transduction, and metabolic pathways. Moreover, several pathways were significantly enriched amongst the differentially expressed genes (DEGs) in ST genotypes, such as phenylpropanoid biosynthesis, plant-pathogen interaction, isoquinoline alkaloid biosynthesis, and tyrosine metabolism. One hundred seventeen DEGs were common to various stages of both genotypes, identified as core salt-responsive genes, including some transcription factor members, like *MYB*, *WRKY* and *NAC*, and some plant hormone signal transduction related genes, like *PYL*, *PP2C* and *TIFY10A*, which play an important role in the adaptation to salt conditions of this species. The expression patterns of 21 DEGs were detected by quantitative real-time PCR (qRT-PCR) and confirmed the reliability of the RNA-Seq results.

**Conclusions:**

We identified candidate genes involved in salt tolerance in quinoa, as well as some DEGs exclusively expressed in ST genotype. The DEGs common to both genotypes under salt stress may be the key genes for quinoa to adapt to salinity environment. These candidate genes regulate salt tolerance primarily by participating in reactive oxygen species (ROS) scavenging system, protein kinases biosynthesis, plant hormone signal transduction and other important biological processes. These findings provide theoretical basis for further understanding the regulation mechanism underlying salt tolerance network of quinoa, as well establish foundation for improving its tolerance to salinity in future breeding programs.

**Supplementary Information:**

The online version contains supplementary material available at 10.1186/s12870-020-02753-1.

## Background

Soil salinity is a major abiotic stress to modern agriculture in arid and semi-arid regions, severely affecting seed germination, plant growth and development, and crop productivity [1, 2]. It is estimated that approximately 800 million hectares, accounting for 6.5% of the total land area of the world, are impacted by salt stress [3]. Worse still, global warming and irrational exploitation and utilization of land have been accelerating soil salinization, which will lead to the loss of half of the arable land by 2050 [4, 5]. The expansion of soil salinization together with the growing world population have put great pressures on agricultural development [6]. Therefore, it is extremely urgent for us to screen salt-tolerant varieties or improve the salt tolerance of plants by genetic engineering to address the problem of salinity.

High salinity disrupts homeostasis in water potential and ion distribution, causing hyperosmotic stress, ion imbalance and oxidative damage, which eventually threatens plant life [7, 8]. The reduced leaf size, stomatal closure, photosynthesis inhibition and decreased biomass are response strategies for coping with salt stress [9]. To survive against salt stress, plants have evolved a series of response mechanisms involving complex physiological traits, metabolic and signaling pathways, and molecular networks to enhance their salt tolerance [10, 11]. Obvious variation in salinity tolerance of different species and varieties is observed. Generally, Salt-tolerant plants maintain a high K^+^/Na^+^ ratio in cytoplasm, which is very important for cell function, and these ions regulate the physiological and developmental processes of plants [12]. Plants respond and adapt to salt stress mainly by multiple biosynthetic and signaling pathways regulated by gene expression [13]. At the molecular level, many key genes related to salt tolerance have been identified and their regulation mechanisms are analyzed exhaustively. *AtHKT1* (high-affinity potassium transporter 1) drives adaptation of *Arabidopsis thaliana* to salinity by limiting the root-to-shoot Na^+^ transportation [14]. Overexpression of four *SOS1* (salt overly sensitive 1) genes improves the salinity tolerance of transgenic chrysanthemum plants [15]. *ZFP179* (zinc-finger protein 179) enhances salt tolerance in rice by modulating oxidative stress responses [16]. *SlCBL10* (calcineurin B-like protein 10) mediates salt tolerance in tomato by regulating Na^+^ and Ca^2+^ fluxes in the vacuole [17]. Overexpression of *GsPRX9* (a peroxidase gene) enhances the tolerance to salt stress and antioxidant response in soybean [18]. Ectopic expression of *PtCYP714A3* (a cytochrome P450 monooxygenase gene) from *Populus trichocarpa* retards shoot growth and confers resistance to salt stress in transgenic rice [19]. Plasma membrane bound *SbNHXLP* (a Na^+^/H^+^ antiporter-like protein) from sorghum involves in Na^+^ exclusion, maintains ion homeostasis and alleviates NaCl stress in transgenic tomato, and more notably, it increases the fruit yield of tomato [20]. Besides, various transcription factors (TFs) had been shown to participate in salt stress response, such as NAC [21], basic leucine zipper (bZIP) [22], APETALA2/ethylene response factor (AP2/ERF) [23], WRKY [24], basic helix-loop-helix (bHLH) [25] and MYB [26]. They regulate the expression levels of multiple downstream genes, which might ultimately affect salt tolerance of plants. Plant hormones are significant regulators of complicated developmental processes and stress-responsive signaling networks [27], and the role of abscisic acid (ABA) [28], ethylene (ET) [29], auxin [30], jasmonate (JA) [31] and brassinosteroid (BR) [32] or their key components in plant response to salt stress have been well studied. These researches also demonstrated that different biological pathways and metabolic processes manipulated by salt-induced genes are interrelated and function together to resist salt stress.

Quinoa (*Chenopodium quinoa* Willd.) is an annual broad-leaved herbaceous plant belonging to the Amaranthaceae family, originated from the Andean region of South America [33]. Quinoa is a pseudocereal crop with high nutritional value, rich in essential amino acids, minerals, dietary fibers, vitamins, and antioxidant components [34]. High tolerance to drought, frost and salinity stresses enable quinoa to adapt to various extreme climate and soil conditions [35–37], which is of great practical significance to develop water-saving agriculture, raise the utilization efficiency of dry land, improve the coastal saline-alkaline soil and maintain the diversity of agricultural ecosystem. Due to the outstanding grain nutritional quality and strong tolerance to various abiotic stresses, the potential crop quinoa has attracted worldwide attention, and the Food and Agriculture Organization of the United nations has declared 2013 the International Year of the Quinoa [38]. Being domesticated as a staple food for aboriginal inhabitants of Andes since 7000 years ago, quinoa diversity can be classified into five major ecotypes: salares, inter-Andean valleys, highlands, yungas and coastal lowlands [39], and the salares landraces are deemed to have the highest salinity tolerance [40]. Until now, more than 16,400 accessions of quinoa and its wild relatives have been conserved in 59 gene banks across 30 countries [41]. However, the salt tolerance of different quinoa varieties varies greatly, some even can grow at salt concentrations similar to those of seawater, while some are relatively weak [42]. Although the tolerance to salinity has become the focus of quinoa research recently, it is mainly about agronomic performance and physiological responses. Identifying salt-tolerant genes is a vital ingredient of salt-tolerant crop breeding through genetic engineering [43]. Numerous genes and signal pathways related to the response to high salinity have been identified in many plants, but only a few have been documented in quinoa. The publication of high-quality reference genome of quinoa and the completion of related transcriptome research facilitated the identification of salt-responsive genes and provided insights into accelerating the genetic improvement of quinoa [44–46]. Some candidate salt tolerance genes predicted to encode transmembrane proteins were identified in quinoa by integrating physiological data, RNA-seq, and single nucleotide polymorphism (SNP) analyses [45]. And transcriptome sequencing was also performed on epidermal bladder cells, a cell structure homologous to trichomes, to unveil the mechanisms of quinoa salinity tolerance [46]. Nevertheless, little effort has been made by far to elucidate the molecular mechanisms underlying the adaptation to salt stress in quinoa.

In the present study, a comparative transcriptome profiling of roots was performed using RNA-Seq to uncover the differential response under salinity stress at the molecular level in two contrasting quinoa genotypes: salt-tolerant QQ056 and salt-sensitive 37TES. We analyzed the differential expressed genes (DEGs) in the two genotypes at each salt treatment time point (0.5, 2, and 24 h). The core DEGs common to both genotypes at three time points under salt stress were identified and their expression patterns were validated by quantitative real-time PCR (qRT-PCR). The possible biological processes and metabolic pathways involved by these salt-responsive genes were also analyzed, providing valuable insights into revealing the molecular mechanisms underlying the adaptation to salt stress, as well developing salt-tolerant quinoa varieties in future breeding programs.

## Results

### Phenotypic differences between salt-tolerant and salt-sensitive genotypes under salinity stress

**Fig. 1:**
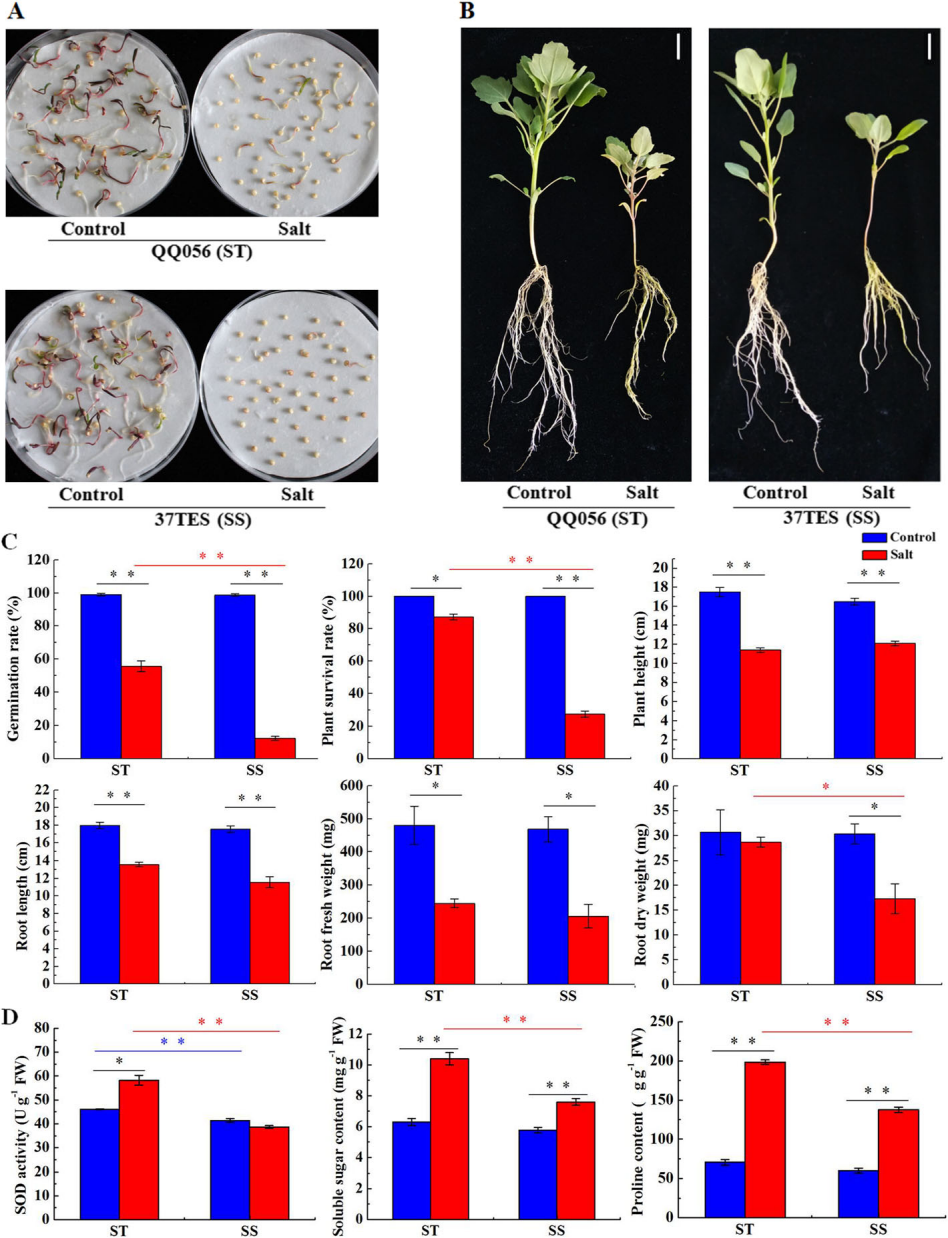
Morphological changes of salt-tolerant (ST) and salt-sensitive (SS) genotypes under salinity stress. **a** Germination of ST and SS under control and 300 mM NaCl conditions. **b** Phenotypic changes of ST and SS when 28-day-old seedlings were exposed to 300 mM NaCl for 14 d. **c** Changes in seed germination rate, plant survival rate, plant height, root length, root fresh weight and root dry weight of ST and SS under control and NaCl treatments. **d** Comparative analysis of SOD activity, soluble sugar content and proline content between different groups and treatments. Scale bars = 2 cm. Multiple comparisons were performed with significant difference (*P < 0.05, **P < 0.01, one-way ANOVA and Tukey’s test; Error bars represent SD)

Compared with control, seed germination rates of QQ056 (salt-tolerant, ST) and 37TES (salt-sensitive, SS) under 300 mM NaCl treatment were both significantly decreased, but the descender of SS was obviously higher than ST (Fig. 1a and c). And the germination rate of ST was significantly higher than SS under salt stress. Likewise, the ability to resist salinity stress of SS seedlings was also weaker than ST. The two contrasting genotypes both showed reduced growth, leaf chlorosis, delicate seedlings and decreased leaf number in response to salt stress, but ST was less damaged than SS (Fig. 1b). Although no obvious differences in plant survival rate, plant height, root length, root fresh weight and root dry weight were observed between the two genotypes under the control condition, the reduction in plant survival rate and root dry weight of ST genotype were less significant than SS and these two indexes of ST were both significantly higher than SS under salt treatment (Fig. 1c). Taken together, ST genotype possesses better salt tolerance than SS genotype. Compared with control, soluble sugar content and proline content of the two genotypes were both significantly increased, the superoxide dismutase (SOD) activity was significantly rised in ST but slightly declined in SS (Fig. 1d). Except SOD activity, there were no significant differences in soluble sugar content and proline content of both genotypes under control condition. But all of the three physiological indexes of ST were significatly higher than SS under salt treatment.

### RNA sequencing and transcriptome assembly

The RNA samples from the roots of 28-day-old ST and SS seedlings under the control and salt-treated (300 mM NaCl, 0.5, 2, and 24 h) conditions were used for Illumina Genome Analyzer deep sequencing. On average, 41,470,921 raw reads were generated, and 38,510,203 clean reads were obtained after cleaning and quality checks from each sample (Table S1). The average clean rate can be high as 92.86%, demonstrating the high quality of the sequencing results. Approximately 85.30% of the reads were mapped to the quinoa reference genome, 33,182,448 reads were mapped to unique regions and 2,192,951 reads were mapped to multiple regions (Table S1).

### Identification and analysis of DEGs under salt treatment

**Fig. 2:**
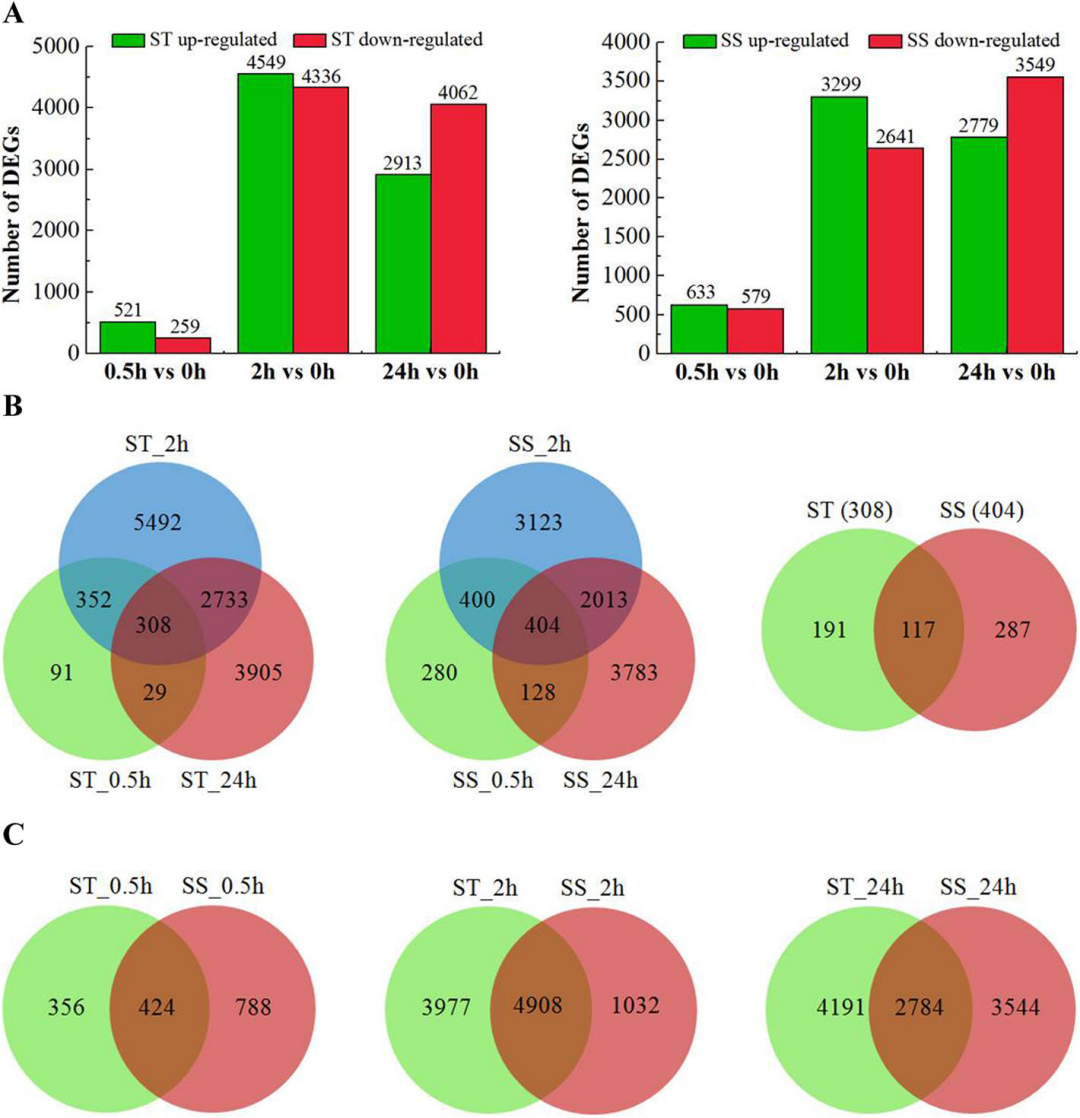
Differentially expressed genes (DEGs) in ST and SS under salt stress. **a** Number of DEGs in ST and SS at different salt stress time points. **b** Venn diagrams of DEGs at different salt stress time points in ST and SS, respectively. **c** Venn diagrams of DEGs between ST and SS at 0.5, 2 and 24 h, respectively

Compared with control, 780/1212, 8885/5940, and 6975/6328 genes were differentially expressed in ST/SS at 0.5, 2, and 24 h, respectively (Fig. 2a). The total number of DEGs was greater in ST than in SS under salt stress. However, the number of up-regulated and down-regulated DEGs in ST and SS at different salt stress time points also existed differences. Among these DEGs, 521/633, 4549/3299, and 2913/2779 were up-regulated in ST/SS at 0.5, 2, and 24 h, respectively, while 259/579, 4336/2641, and 4062/3549 were correspondingly down-regulated (Fig. 2a). Venn-diagram analysis indicated that the salt-responsive genes were genotype specific and time specific, which may account for the differences in salt tolerance of ST and SS genotypes (Fig. 2b and c).

**Fig. 3:**
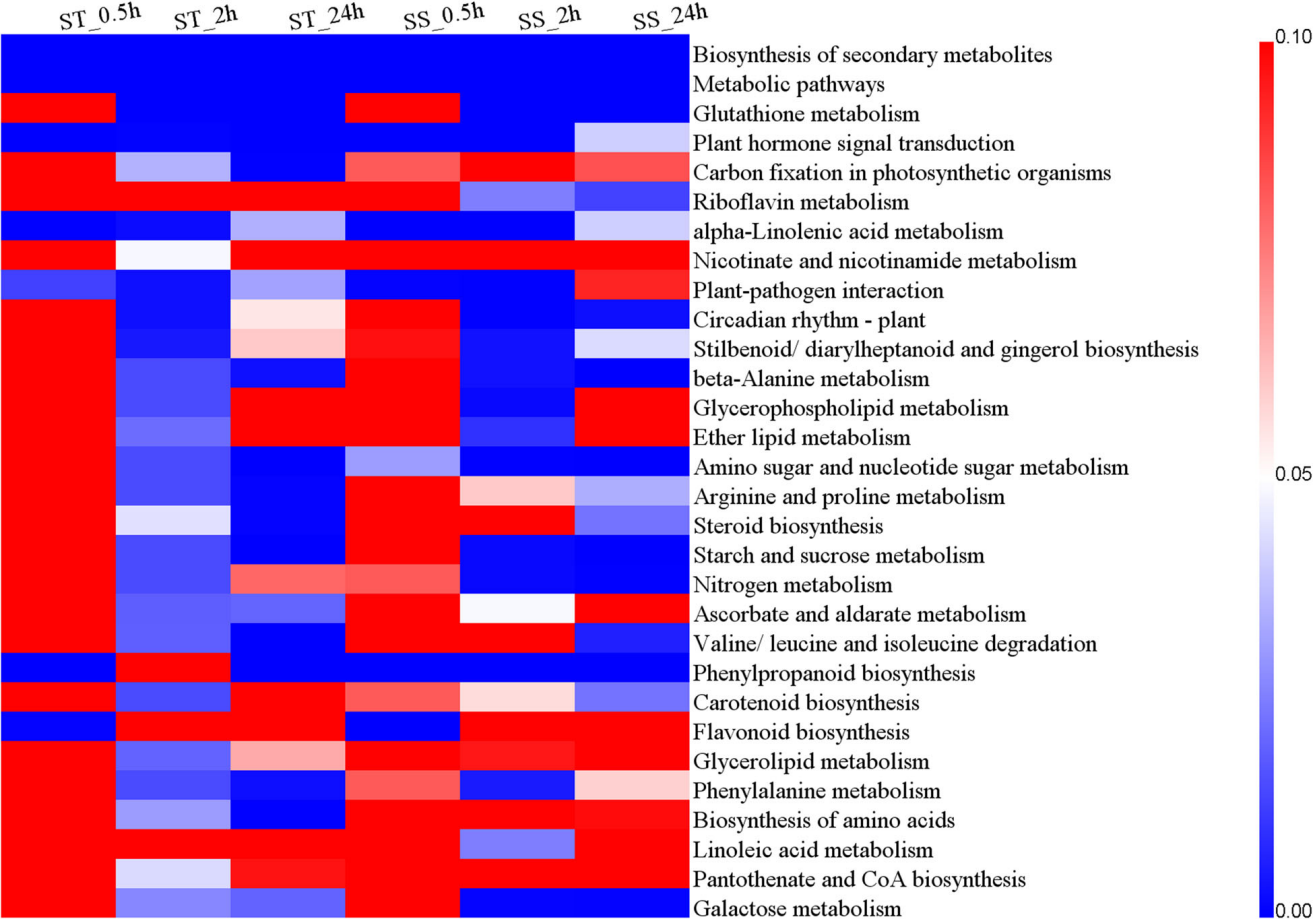
KEGG pathway classification and enrichment analysis of the DEGs in ST and SS at different salt stress time points. Blue color represents significant enrichment, red color represents non-significant enrichment

The KEGG pathway enrichment analysis among all the DEGs were performed to reveal several important salt-related pathways. These pathways mainly involved biosynthesis of secondary metabolites, amino acid metabolism, vitamin metabolism, lipid metabolism, plant hormone signal transduction, carotenoid biosynthesis, and carbohydrate metabolism (Table S2). Among these, biosynthesis of secondary metabolites, metabolic pathways, plant hormone signal transduction and alpha-Linolenic acid metabolism were significantly enriched in both genotypes at each salt stress time point (Fig. 3). The significant enrichment of plant hormone signal transduction revealed the importance of plant hormones in salt stress. Based on 271 background genes in plant hormone signal transduction in quinoa, 25/30, 133/106, and 108/82 DEGs were enriched in this pathway in ST/SS at 0.5, 2, and 24 h, respectively (Table S2). In addition, some pathways were preferably enriched in ST or SS at all salt stress time points, such as plant-pathogen interaction in ST, amino sugar and nucleotide sugar metabolism and phenylpropanoid biosynthesis in SS, suggesting the differences in response mechanisms to salt stress of the two genotypes (Fig. 3).

**Fig. 4:**
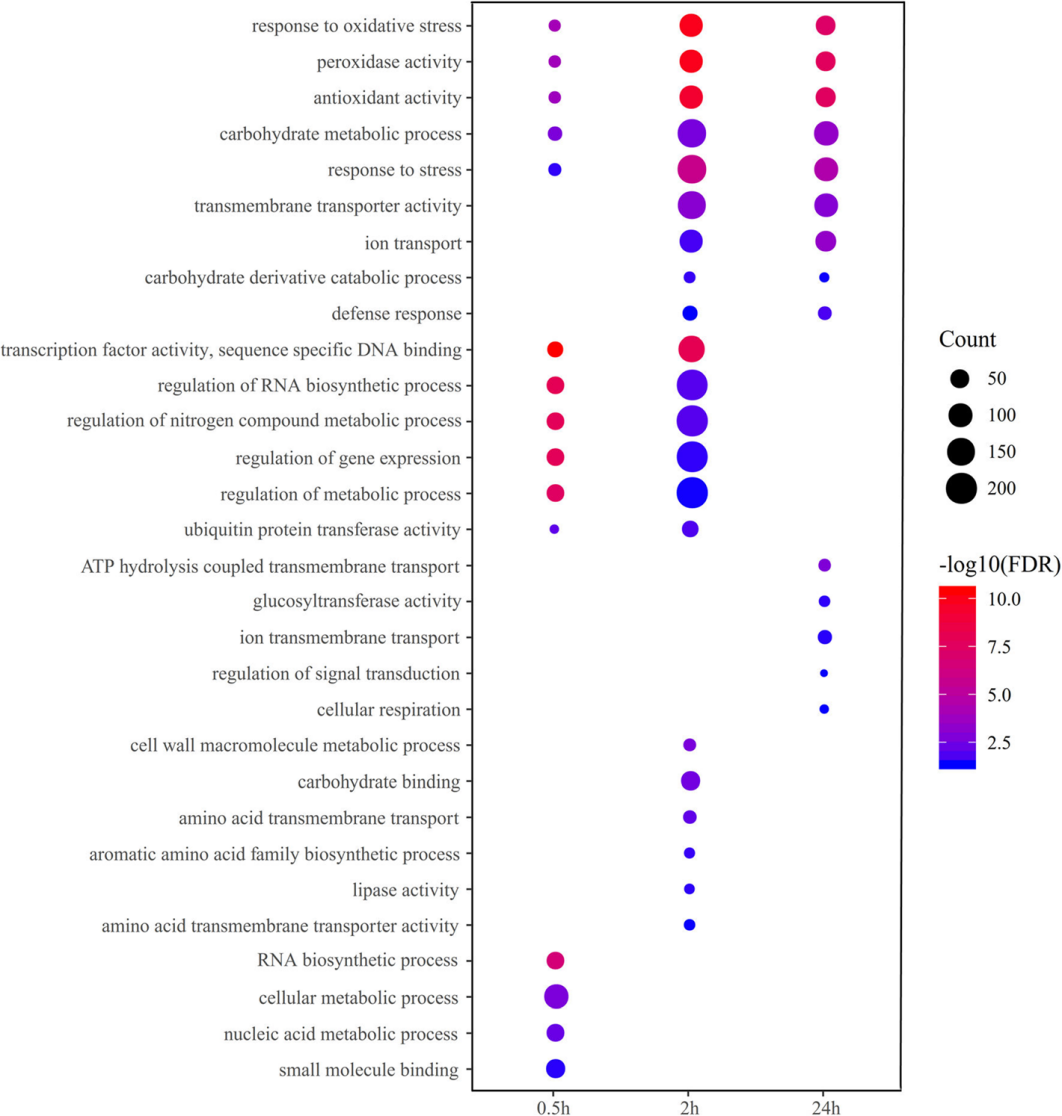
Overrepresented GO terms common in both quinoa genotypes at different time points. X-axis, salt stress treatment time; Y-axis, enriched GO terms

GO enrichment analysis was conducted to characterize the biological functions of the DEGs. The results indicated that several GO terms were significantly enriched in both genotypes at all salt stress time points, including response to oxidative stress, oxidation-reduction process, oxidoreductase activity, and antioxidant activity, which have been proved to be related to salt tolerance in plants (Tables S3 and S4). At 0.5 h of 300 mM NaCl stress, transcription factor activity, sequence specific DNA binding, regulation of RNA biosynthetic process, and regulation of gene expression were significantly enriched in both genotypes, while the GO terms response to oxidative stress, peroxidase avtivity, and antioxidant activity were significantly enriched at 2 h and 24 h of salt stress (Fig. 4). These suggested that the initial stage of response to salt stress mainly involved the transcription activation or expression of some important genes with molecular functions, with the extension of salt treatment time some biological processes were induced. Meanwhile, the important role of transcription factors in response to salt stress was also revealed.

### Analysis of the DEGs exclusively in ST genotype in response to salt stress

A total of 308 and 404 DEGs were commonly identified at all sampling time points in ST and SS, respectively, whereas 191 and 287 DEGs were exclusively found in ST and SS under NaCl treatment, respectively (Fig. 2b). Further analysis showed that 87 genes were up-regulated in ST among the 191 DEGs above, including calcium-dependent protein kinase, ethylene-responsive transcription factors, heat shock factor protein, UDP-glycosyltransferase, transcription factors WRKY and MYB, which might be candidate genes for improved salt tolerance in ST (Table S5). The most representative GO categories amongst the 191 DEGs exclusively in ST were biological processes related to response to oxidative stress, response to stimulus and response to stress, molecular functions related to peroxidase activity, antioxidant activity and sequence-specific DNA binding, cellular components related to extracellular region and cell wall (Table S6). The significantly enriched KEGG pathways included phenylpropanoid biosynthesis, biosynthesis of secondary metabolites and metabolic pathways (Table S7). Eleven genes were enriched in phenylpropanoid biosynthesis, most of which were related to peroxidases.

### Common DEGs between ST and SS under salt stress

In spite of the exclusively expressed differential genes in each genotype, 117 DEGs were common to various stages of both genotypes (Fig. 2b). These genes (83 up- and 34 down-regulated) were constitutively active and were called core salt-responsive genes despite different salt tolerance levels in quinoa, including many peroxidases (PODs), protein phosphatase 2Cs (PP2Cs), zinc finger proteins (ZATs), F-box proteins and plasma membrane ATPase (Table S8). Especially, several transcription factors were also included, such as WRKY, NAC and MYB, suggesting their important roles in transcription regulation contributing to salt tolerance of quinoa.

**Fig. 5:**
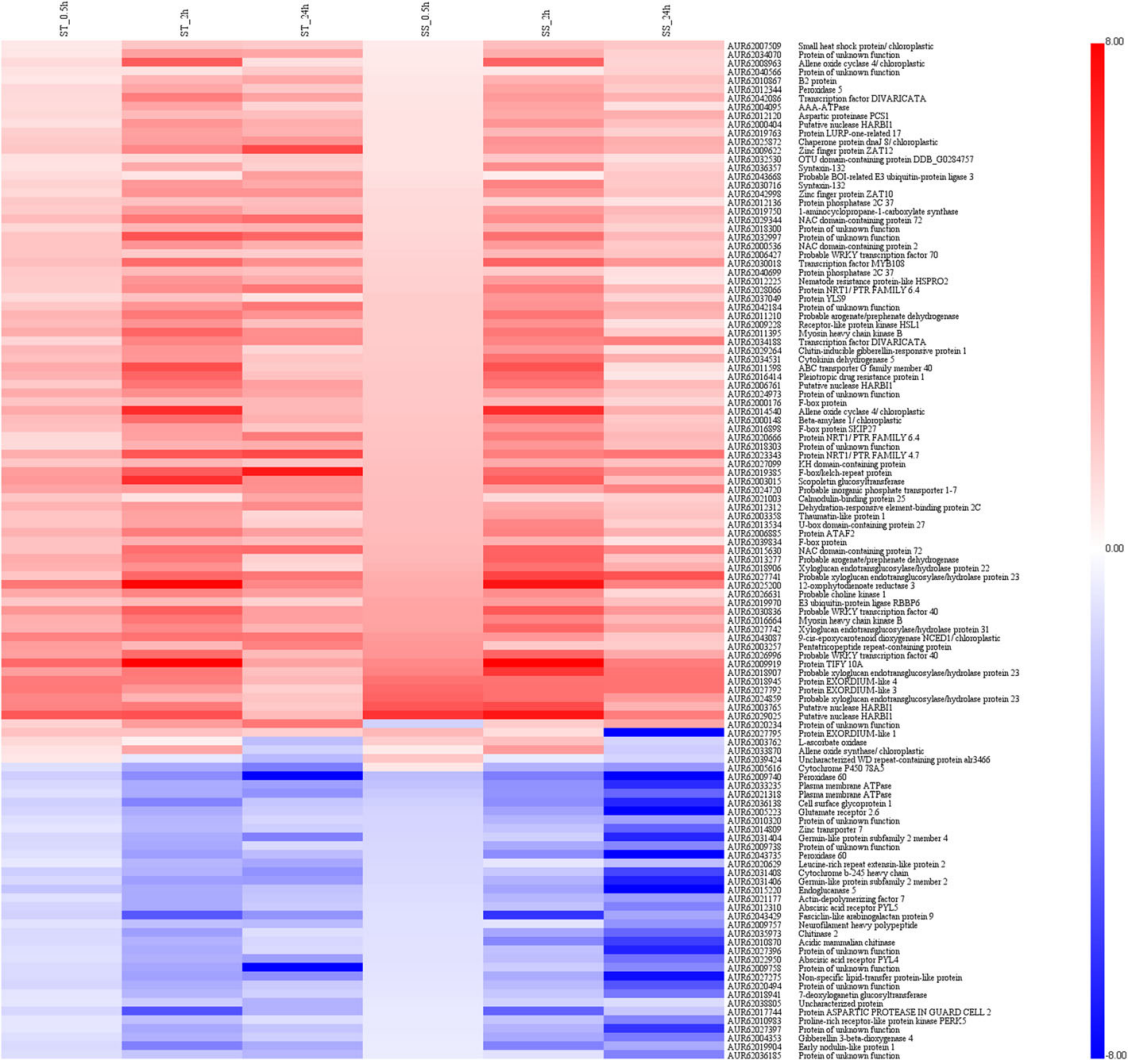
Heatmap of the 117 core DEGs in ST and SS at different salt stress time points. X-axis, samples; Y-axis, differentially expressed gene names with annotations

In addition, the expression patterns of the constitutively active genes were analyzed in both genotypes under salt stress. As shown in Fig. 5, the temporal and spatial expression patterns of 117 core DEGs were presented by a heatmap. Although there was a big difference in salt tolerance between the two genotypes, these DEGs exhibited similar expression patterns, indicating that these genes were commonly induced in this species in response to salinity stress.

### GO enrichment analysis of common DEGs of ST and SS in response to salt stress

**Fig. 6:**
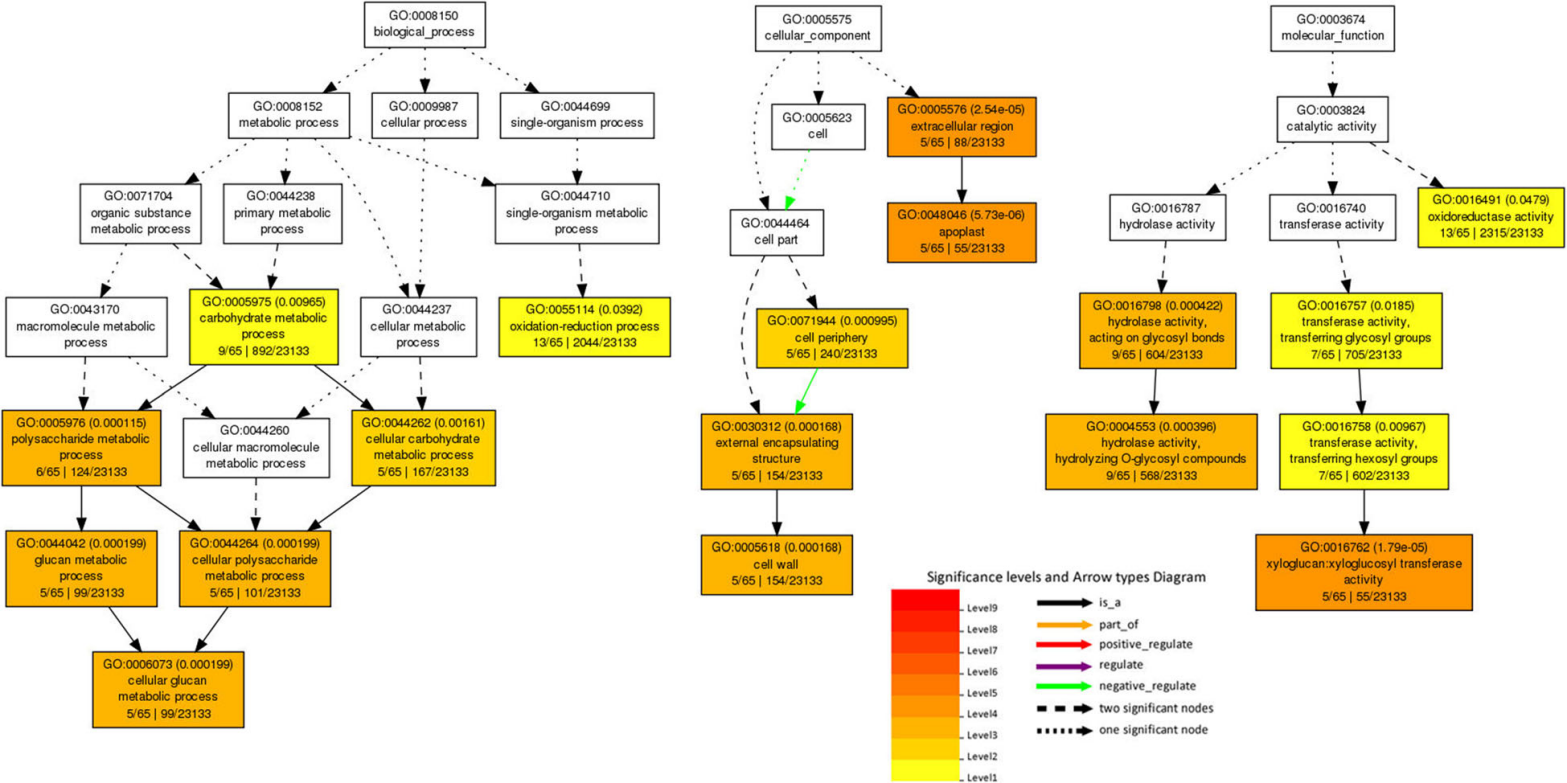
Enriched GO terms for core genes in response to salt stress. The significant GO terms are in colored boxes, and non-significant terms are in white boxes

Based on GO enrichment analysis, the 117 core genes were categorized into three GO ontologies and 32 terms (Fig. 6). Many genes were significantly enriched in biological processes concerned with polysaccharide metabolic process, cellular glucan metabolic process, cellular polysaccharide metabolic process, glucan metabolic process, cellular carbohydrate metabolic process, carbohydrate metabolic process, and oxidation-reduction process (Fig. 6 and Table S9). Furthermore, some GO categories were significantly enriched in molecular functions amongst the DEGs, including xyloglucan:xyloglucosyl transferase activity, hydrolase activity, hydrolyzing O-glycosyl compounds, hydrolase activity, acting on glycosyl bonds, transferase activity, transferring hexosyl groups, transferase activity, transferring glycosyl groups, and oxidoreductase activity (Fig. 6 and Table S9). Hence, the salt resistance process is very complicated in quinoa.

### KEGG pathway enrichment analysis of common DEGs of ST and SS in response to salt stress

The core genes were aligned with the KEGG database and were assigned to 16 KEGG pathways (Table S10). The significantly enriched pathways were alpha-Linolenic acid metabolism, biosynthesis of secondary metabolites, phenylpropanoid biosynthesis, plant hormone signal transduction, metabolic pathways, and SNARE interactions in vesicular transport (Table S10). The metabolic pathways exhibited the largest number of DEGs, indicating many biochemical reactions were activated to enhance the adaptation to salt environment. Four DEGs were enriched in phenylpropanoid biosynthesis, three of which were identified as PODs.

**Fig. 7:**
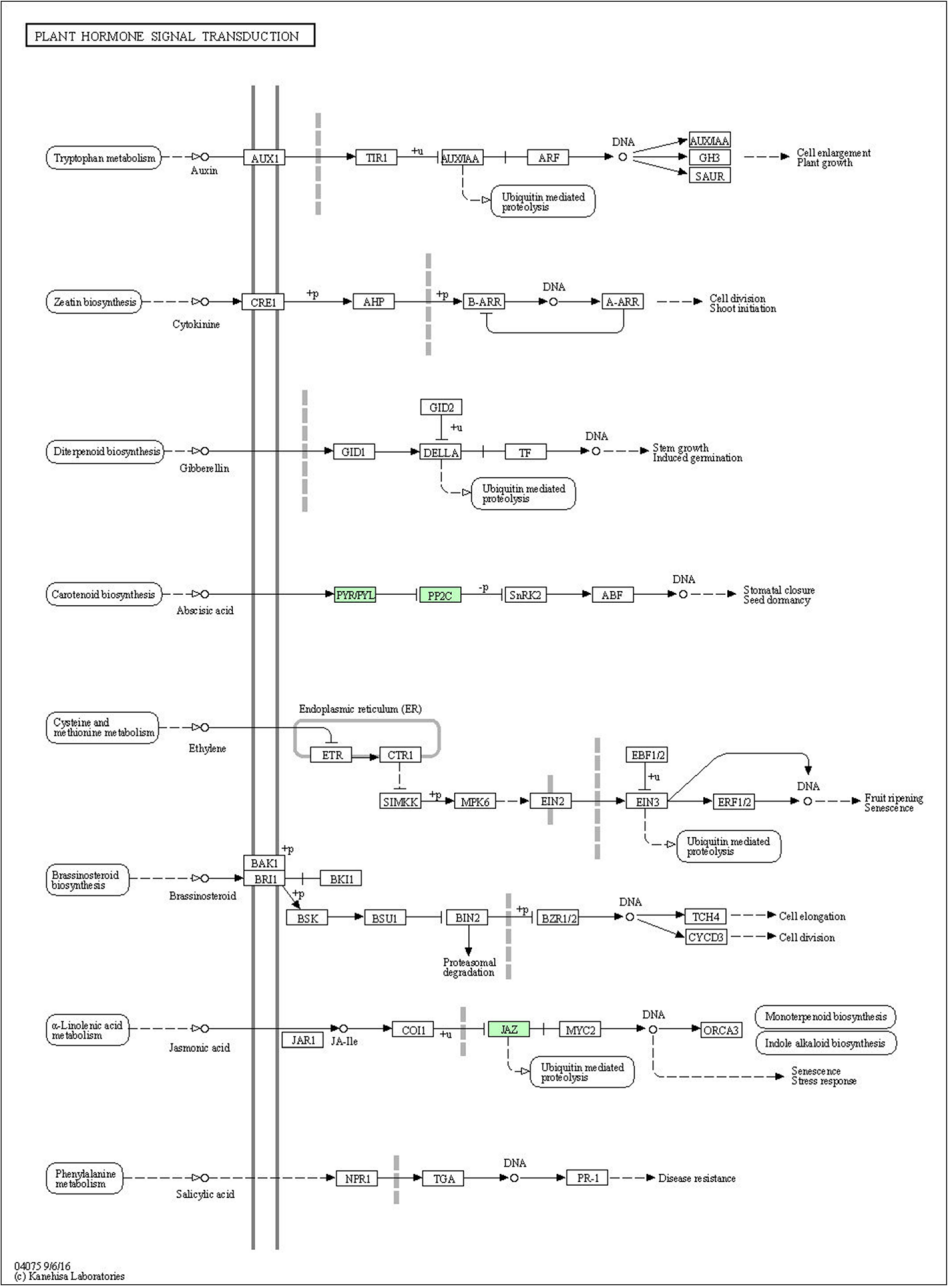
Overview of plant hormone signal transduction pathway in quinoa. This diagram represents the signaling pathway of eight plant hormones in quinoa. Through the proteins in the light green frames we can specifically know which hormonal pathways are involved among the common DEGs in both quinoa genotypes under salt stress

Five differentially expressed transcripts were predicted to participate in plant hormone signal transduction, including two PP2Cs (AUR62012136, AUR62040699), one TIFY10A (AUR62009919), and two ABA receptor PYLs (AUR62012310, AUR62022950) (Tables S8 and S10). PP2C and PYL are important participants in ABA signaling pathway, their involvements eventually cause stomatal closure to respond to salt stress (Fig. 7). The JA-responsive protein TIFY10A can immediately induce ubiquitin mediated proteolysis or indirectly promote monoterpenoid biosynthesis and indole alkaloid biosynthesis to respond to stimulus (Fig. 7). The *PP2Cs* and *TIFY10A* were positively regulated in both genotypes under 300 mM NaCl treatment, while the *PYLs* were negatively regulated, indicating their different roles in the adaptation to high salinity. Meanwhile, it also revealed the significance of plant hormones in resistance to salt stress in quinoa.

### Validation of RNA-Seq data by qRT-PCR analysis

**Fig. 8:**
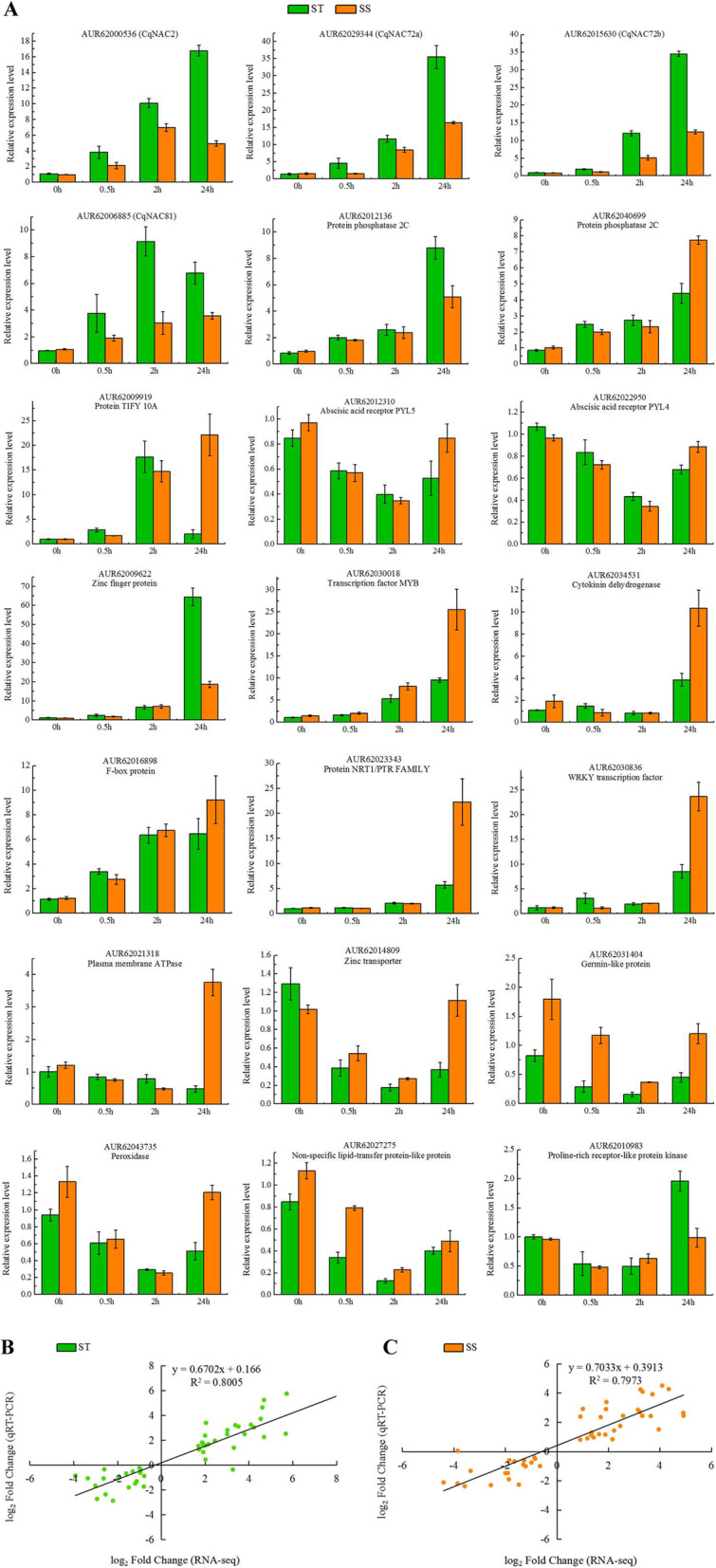
qRT-PCR validation of expression profiles obtained by RNA-seq in ST and SS under salt stress. **a** qRT-PCR analysis of 21 selected genes. The Y-axis represents the relative expression levels, the X-axis shows the time of 300 mM NaCl treatment in both genotypes. The correlation of log_2_ Fold Change obtained by qRT-PCR (Y-axis) and RNA-seq (X-axis) in ST (**b**) and SS (**c**)

To further validate the expression data of RNA-Seq, qRT-PCR was performed on the same RNA samples originally used for next-generation sequencing. Twenty-one DEGs common in both genotypes under salt stress were selected for qRT-PCR analysis, including six transcription factors (four *NACs*, one *MYB* and one *WRKY*), five plant hormone-related genes (two *PP2Cs*, one *TIFY10A* and two *PYLs*) and other ten key genes (Fig. 8a). Among these, 13 were up-regulated and 8 were down-regulated (Table S8). The specific primers are listed in Table S11. To compare the expression data between RNA-Seq and qRT-PCR, the relative expression level was transformed to log_2_ Fold Change. The qRT-PCR results were strongly correlated with RNA-Seq data both in ST (*R*^*2*^ = 0.8005) (Fig. 8b) and SS (*R*^*2*^ = 0.7973) (Fig. 8c), demonstrating the reliability of the RNA-Seq expression profile in this study.

## Discussion

Soil salinity is a major environmental stress and has become a prime concern for global crop production and food security. Although quinoa is more salt tolerant than other crops, high salinity above a certain concentration threshold significantly affects its yield and quality. Thus it is necessary to identify some important salt response mechanisms and salt tolerance mechanisms to develop new quinoa varieties with high and stable yield under high salt environments. In this study, we performed transcriptomic analysis in quinoa seedlings of two contrasting genotypes at 0, 0.5, 2, and 24 h of 300 mM NaCl stress, and the DEGs especially common in both genotypes and exclusively in ST were identified and further analyzed, which could provide insights into the candidate genes and metabolic pathways underlying salt tolerance in quinoa. Among these DEGs, the genes related to ROS scavenging system, protein kinases, plant hormone signal transduction and transcription factors were prevalent and dominant.

### ROS scavenging system under salt stress

Salt stress can dramatically increase the production of ROS [47], causing oxidative damage to cellular structures and eventually leading to programmed cell death [48]. The balance between ROS generation and quenching is prerequisite for the cell survival which is maintained by the complex anti-oxidative system [49]. In the adaptation to external environment stresses, plants have developed various non-enzymatic and enzymatic ROS detoxification mechanisms. Antioxidant enzymes such as POD, superoxide dismutase (SOD), ascorbate peroxidase (APX),and catalase (CAT) play important roles in scavenging ROS and can maintain the ROS at low levels under salinity stress [50]. In this study, a great number of DEGs related to oxidoreductase activity and oxidation-reduction process were identified in both genotypes, implying that oxidative stress could be induced by a short period of salt stress. POD activity, antioxidant activity, together with another two GO terms mentioned above were all significantly enriched amongst the DEGs exclusively in ST, indicating their importance in improving salt tolerance. POD, as an important antioxidant enzyme in ROS scavenging system, can be encoded by certain genes. We found *POD5* (*AUR62012344*), one coding gene of POD, was distinctly up-regulated in both genotypes under salt stress, suggesting that ROS scavenging system has important functions in protecting quinoa from salt stress, in spite of salt tolerance level.

### Protein kinases involved in salt stress

Protein kinases play crucial roles in plant development and environmental stress responses. Recptor-like protein kinases (RLKs) are membrane-anchored proteins, often carrying extracellular leucine-rich repeats (LRRs), which act as sensors and receptors mediating signaling transduction [51]. The overexpression of *AtRPK1* enhanced salt tolerance in transgenic Arabidopsis [52]. Here, one *RPK* (*AUR62009228*) and five *LRR-RPKs* (*AUR62039001*, *AUR62021617*, *AUR62018804*, *AUR62007198* and *AUR62040226*) were identified as up-regulated DEGs in both genotypes and exclusively in ST, respectively, indicating their important functions in quinoa salt stress resistance. Another subclass of the receptor-like kinases is proline-rich, extension-like receptor-like kinases (PERKs), which are a group of proteins that act as sensors/receptors at the cell wall. Especially, they can cause changes in plant cell wall when exposed to environmental stresses, and inhibit root growth [53]. We found one differentially expressed *PERK* gene (*AUR62010983*) both in ST and SS was strongly down-regulated under salt treatment, implicating its probable negative regulation function in the adaptation to saline environment. Calcium-dependent protein kinases (CDPKs) are indispensable for modulating abiotic stress tolerance, including drought, cold, heat and salinity [54]. In this study, three *CDPKs* (*AUR62029467*, *AUR62024429* and *AUR62026022*) were found to be exclusively and dramatically up-regulated in ST in response to salt stress, suggesting their great potential for improving the salt tolerance of quinoa.

### Plant hormone signal transduction under salt stress

Plant hormones play pivotal roles in enhancing resistance to environmental stresses [55]. In this study, a large number of DEGs were significantly enriched in the pathway of plant hormone signal transduction in ST and SS at different time points of salt treatment. ABA is an essential messenger for plants to adapt to abiotic stresses [56]. There are three important components of ABA signaling: ABA receptors PYR/PYL/RCARs, negative regulators PP2Cs and positive regulators SnRKs. Previous studies demonstrated that *TaPP2C1* confers resistance to salt stress in transgenic tobacco through activating the antioxidant system and ABA-independent gene transcription process [28]. Similarly, several *PP2Cs* were found highly up-regulated in both quinoa genotypes (two *PP2Cs*) and exclusively in ST (four *PP2Cs*) under salt stress, indicating their negative regulation roles in ABA signaling pathway but positive roles in salt resistance. TIFY proteins, acting as repressors of JA responses, play an important role in plant development and response to abiotic stresses [57]. In the current study, one differentially expressed gene *TIFY10A* common in ST and SS was strongly induced in response to salt stress, which might protect quinoa against salt stress by regulating JA signaling pathway. Furthermore, we also found one gibberellin (GA)-related gene *GA 3-beta-dioxygenase* (*GA3OX4*) (*AUR62004353*), considered involved in GA biosynthesis pathway, was strongly inhibited in quinoa under salt treatment, implying that GA signal may also participate in the salt stress signal pathway of quinoa. However, further efforts are still needed to elucidate the molecular mechanisms of these plant hormone-related genes.

### Transcription factors related to salt stress in quinoa

Transcription factors such as NAC, bZIP, AP2/ERF, WRKY, MYB and bHLH regulated the expression of salt-responsive genes and ultimately determined the salt tolerance level of plants [21–26, 58]. Twelve TFs differentially expressed in both genotypes under NaCl treatment were indentified in the transcriptome analysis, including three *MYBs* (*AUR62042086*, *AUR62034188* and *AUR62030018*), four *NACs* (*AUR62000536*, *AUR62029344*, *AUR62015630* and *AUR62006885*), three *WRKYs* (*AUR62030836*, *AUR62026996* and *AUR62006427*), and two *ZFPs* (*AUR62042998* and *AUR62009622*). It has been shown that two *ZFP* members *Zat10* and *Zat12* could be induced by salinity stress in Arabidopsis [59, 60], while the two homologous genes in quinoa were highly up-regulated as well in this study, which indicated their potential roles in salt stress. Apart from four WRKYs and four MYBs, one *ERF* (*AUR62028234*) and one *bZIP* (*AUR62010368*) were also found in the DEGs exclusively in ST genotype, all of which were strongly induced in response to salt stress, suggesting their probable functions in improved salt tolerance. Some *ERFs* conferred tolerance to high salinity through modulating ABA and ET levels [61], while some were through amplifying the ROS-activated mitogen-activated protein kinase (MAPK) cascade signal during the initial phase of salt stress [62]. These results showed that salt tolerance is an extremely complicated process, involved in various biological processes and metabolic pathways, the transcriptional networks and interactions of regulatory genes deserve further research.

## Conclusions

In the present study, a lot of candidate genes underlying salt tolerance in quinoa were identified through transcriptome analysis, which might be of great value in salt tolerant breeding of quinoa in the future. Most of these genes, including some TFs, are involved in ROS scavenging, plant hormone signal transduction, biosynthesis of secondary metabolites, and metabolic pathways, suggesting that salt tolerance of quinoa is a complex mechanism. These findings provide valuable information for understanding the molecular regulatory network of quinoa salt tolerance, and provide genetic resources and theoretical basis for the subsequent research on gene function and salt tolerance improvement of quinoa.

## Methods

### Plant materials and NaCl treatments

Two quinoa accessions of salt-tolerant QQ056 (ST) and salt-sensitive 37TES (SS) from United States Department of Agriculture (USDA) were used for this study. QQ056 originates in Chile with the USDA number PI 584524, and 37TES originates in the United States, New Mexico with the USDA number Ames 13723. The seeds were surface-sterilized with 3% hydrate peroxide for 1 min and rinsed several times using sterile water, and then germinated at 28 °C under long-day conditions with a photoperiod of 16 h/8 h (day/ night) in a phytotron. Seven days later, the uniform seedling were transferred to half-strength Hoagland nutrient solution. 28-day-old seedlings were treated with 300 mM NaCl. After exposure for 0 (control), 0.5, 2, and 24 h, the root samples were frozen in liquid nitrogen and stored at − 80 °C for transcriptome sequencing and qRT-PCR validation. Phenotypic changes were observed after 14 d of NaCl treatment. Each treatment was set three biological replicates.

### Phenotypic characterization

The effect of salt stress on seed germination of the two genotypes was designed as follows: Place the sterilized seeds on a double layer of filter paper with distilled water (control) and 300 mM NaCl in an illumination incubator at 28 °C for 7 d, respectively, and then recorded seed germination rate. At the seedling stage, after 14 d of NaCl treatment stated before, plant height, root length, root fresh weight and root dry weight under control and salt stress conditions were measured to demonstrate phenotypic changes. Meanwhile, the SOD activity, soluble sugar content and proline content were also determinated by the method of Yang et al. [63]. 28-day-old seedlings were exposed to salt stress for 28 d, and then plant survival rate was recorded. The software SPSS Statistics 17 was used to perform statistical analysis.

### RNA isolation, library construction and transcriptome sequencing

Total RNA was isolated using a RNAprep Pure Plant Kit and treated with DNase I (Tiangen, Beijing, China) according to manufacturer’s instructions. RNA concentrations and quality were determined by a Thermo 2000 Bioanalyzer with a RNA NanoDrop (Thermo Scientific, USA). Library preparation for RNA-Seq was conducted using a TruSeq Stranded mRNA LT Sample Prep Kit (Illumina, Cat. RS-122-2101, USA) according to manufacturer’s protocol. And the final libraries were quantified by QuantiFluor dsDNA System (Promega, USA) and sequenced on an Illumina HiSeq 2500 platform (Illumina Inc. USA) at Berry Genomics Corporation, Beijing, China.

### Reads mapping, sequence assembly and differential expression

After filtering adaptor sequences and low quality bases, the clean reads were mapped to the quinoa genome v1.0 (https://www.ncbi.nlm.nih.gov/genome/?term=quinoa) using Tophat software [64]. The library read alignment files were used as input to the software Cufflinks [65], which assembled the reads into transfrags. Gene expression levels were determined by the fragments per kilobase of transcript per million mapped reads (FPKM). A significant false discovery rate-adjusted *P* value FDR < 0.05, log_2_ Fold Change ≥1 was used as criteria for identifying DEGs.

### Enrichment analysis

GO enrichment analysis of the DEGs was performed using Singular Enrichment Analysis (SER) analysis tool with FDR < 0.05 by agriGO v2.0 [66], and the quinoa genome was set as background. KEGG pathway analysis of the DEGs was carried out by KOBAS 2.0 [67]. Significantly enriched pathways were identified using the Benjamini-Hochberg corrected *P*-value < 0.05. And the overview of plant hormone signal transduction pathways was obtained by KAAS server [68].

### Heatmap with clustering analysis

Hierarchical clustering of the DEGs was analyzed by HemI software [69]. KEGG pathway heatmap was drawn using FDR values. And log_2_ Fold Change values were used in the gene expression heatmap.

### qRT-PCR validation

The same RNA samples for RNA-seq library construction were sued for qRT-PCR analysis. The first strand cDNA was synthesized using the PrimeScript TM RT-PCR Kit (Takara, Japan) according to the manufacturer’s instructions. qRT-PCR was performed with the Real-Time PCR System StepOne version 2.1 (Applied Biosystems, USA) using FastStart Universal SYBR Green Master (Roche, Germany) according to manufacturer’s protocol. *CqEF1a* (*AUR62020767*) was used as an internal control to normalize the gene expression level [70]. Relative expression levels were calculated by 2^-∆∆CT^ method. The primers for qRT-PCR were presented in Table S11.

## Supplementary Information

**Additional file 1: Table S1.** Summary of RNA-Seq results.

**Additional file 2: Table S2.** Significantly enriched KEGG pathways of the DEGs in ST and SS in response to salt stress (Corrected *P*-Value < 0.05).

**Additional file 3: Table S3.** GO enrichment analysis of the DEGs in ST/SS at different time points.

**Additional file 4: Table S4.** Analysis of enriched GO terms common in ST and SS at different time points.

**Additional file 5: Table S5.** List of genes that were exclusively up-regulated in ST genotype at all salt stress time points.

**Additional file 6: Table S6.** GO terms significantly enriched amongst the DEGs exclusively in ST genotype.

**Additional file 7: Table S7.** KEGG pathways analysis of the DEGs exclusively in ST genotype.

**Additional file 8: Table S8.** List of the core genes set involved in quinoa response to salt stress.

**Additional file 9: Table S9.** GO terms significantly enriched amongst the core DEGs in quinoa.

**Additional file 10: Table S10.** Enriched KEGG pathways of the core DEGs in quinoa.

**Additional file 11: Table S11.** Primer sequences for qRT-PCR.

## Data Availability

The datasets during the current study are deposited in the publicly accessible NCBI Sequence Read Archive (SRA) Database as accession numbers (SRR11921127-SRR11921149).

## Abbreviations

ST: Salt-tolerant
SS: Salt-sensitive
DEGs: Differentially expressed genes
GO: Gene ontology
KEGG: Kyoto encyclopedia of genes and genomes
ROS: Reactive oxygen species
POD: Peroxidase
RLKs: Recptor-like protein kinases
CDPKs: Calcium-dependent protein kinases
PP2C: Probable protein phosphatase 2C
ABA: Abscisic acid
JA: Jasmonate
TF: Transcription factor
qRT-PCR: Quantitative real-time PCR
